# Identification of Long Non-Coding RNAs and the Regulatory Network Responsive to Arbuscular Mycorrhizal Fungi Colonization in Maize Roots

**DOI:** 10.3390/ijms20184491

**Published:** 2019-09-11

**Authors:** Guomin Han, Chen Cheng, Yanmei Zheng, Xuewen Wang, Yunjian Xu, Wei Wang, Suwen Zhu, Beijiu Cheng

**Affiliations:** 1 School of Life Sciences, Anhui Agricultural University, Hefei 230036, China; funkii@live.com (C.C.); 18119675403@163.com (Y.Z.); xuyunjian1992@163.com (Y.X.); wangweisys@ahau.edu.cn (W.W.); zhusuwen@126.com (S.Z.); 2 National Engineering Laboratory of Crop Stress Resistance Breeding, Anhui Agricultural University, Hefei 230036, China; 3 Department of Genetics, University of Georgia, Athens, GA 30602, USA; xwwang@uga.edu

**Keywords:** *Zea mays*, arbuscular mycorrhizal fungi, long noncoding RNA, regulatory network

## Abstract

Recently, long noncoding RNAs (lncRNAs) have emerged as vital regulators of many biological processes in animals and plants. However, to our knowledge no investigations on plant lncRNAs which respond to arbuscular mycorrhizal (AM) fungi have been reported thus far. In this study, maize roots colonized with AM fungus were analyzed by strand-specific RNA-Seq to identify AM fungi-responsive lncRNAs and construct an associated regulatory network. A total of 1837 differentially expressed protein coding genes (DEGs) were identified from maize roots with *Rhizophagus irregularis* inoculation. Many AM fungi-responsive genes were homologs to *MtPt4*, *STR*, *STR2*, *MtFatM*, and enriched pathways such as fatty acid biosynthesis, response to phosphate starvation, and nitrogen metabolism are consistent with previous studies. In total, 5941 lncRNAs were identified, of which more than 3000 were new. Of those, 63 lncRNAs were differentially expressed. The putative target genes of differentially expressed lncRNAs (DELs) were mainly related to phosphate ion transmembrane transport, cellular response to potassium ion starvation, and lipid catabolic processes. Regulatory network analysis showed that DELs might be involved in the regulation of bidirectional nutrient exchange between plant and AM fungi as mimicry of microRNA targets. The results of this study can broaden our knowledge on the interaction between plant and AM fungi.

## 1. Introduction

Maize (*Zea mays* L.) is one of the most important crops grown globally for livestock feed, food, and industrial materials [1]. Biotic and abiotic stresses, such as pathogen attacks and lack of mineral nutrients, often limit maize yields [1,2,3,4]. An effective way to improve maize production is to apply artificial chemical fertilizers. Although fertilizers have greatly improved the total yield of crops, massive amounts of fertilizers are often wasted [5,6]. Fertilizers are often washed into water bodies, becoming a major source of agricultural non-point source pollution [6,7]. In addition to chemical fertilizers, agriculturally beneficial microorganisms may also contribute directly or indirectly to crop improvement, increasing fertilizer efficiency [8,9]. Of the microorganisms, arbuscular mycorrhiza (AM) fungi and rhizobia can form a symbiotic association with plants. Rhizobia mainly form a symbiotic association with legumes, while AM fungi (subphylum Glomeromycotina) have widespread symbiosis with land plant species [10,11]. During the AM symbiosis, AM fungi can transfer absorbed nutrients, e.g., 100% phosphorus and 80% nitrogen, to their host plants in exchange for carbohydrates [12]. Both host plants and AM fungi grow better in symbiosis than independently.

The establishment of AM symbiosis is a complex process which is achieved through chemical communication between the plant root and the fungi in the soil [13,14]. Under adverse environments, especially in the absence of available phosphorus, plants release strigolactones from roots as signaling molecules, while AM fungi release myc factors as signaling molecules [10,15]. The two molecules mediate the mutual recognition between the plant roots and fungal hyphae. Myc factors (a mixture of lipochitooligosaccharides), can be perceived by plant LysM receptor-like kinases (e.g., OsCERK1, SlLYK10, LjNFR1, MtLYK3, SYMRK/DMI2) [15]. Then, the factors induce Ca^2+^ spiking, which activates a common symbiosis signaling pathway that is shared by AM symbiosis and root nodulation symbiosis [16]. Nuclear-localized potassium channels (e.g., DMI1, CASTOR, POLLUX), components of the nuclear pore complex (NUP85, NUP133, and NENA), and cyclic nucleotide-gated channels are required for the Ca^2+^ spiking [17,18,19,20,21,22]. A calcium- and calmodulin-dependent serine/threonine protein kinase (CCaMK) and its interacting protein might recognize the calcium signal, and in turn activate the *GRAS* family transcription factor RAM1 [15,23]. As an early transcription factor, RAM1 is able to form complexes with other proteins (e.g., DELLA, NSP2, RAD1, DIP1) and induces the expression of the *WRI5* gene [23]. It is speculated that WRI5 and CBX1 may work together or separately induce the expression of a regulon involved in fatty acid biosynthesis, as well as STR (a ABC transporter), which is involved in the synthesis and transfer of fatty acids to the fungus [10,23,24]. In addition, RAM1 can induce the AM-specific phosphate transporter PT4 and three MIG1 homologs which are engaged in phosphate uptake and the growth of arbuscule-containing cells, respectively [23]. Synergistic expression of the genes mediates the establishment of symbiosis relationship between host plants and AM fungi.

Long noncoding RNAs (lncRNAs) are a class of non-coding RNAs that are longer than 200 nucleotides (nt) [1,25]. Previous investigations showed that lncRNAs play vital roles in regulating diverse biological processes and have emerged as key modulators in gene expression [26,27]. Several lncRNAs are essential in regulation of mRNA processing and transcription mechanisms, e.g., translation, editing, splicing, and localization [27]. Some lncRNAs have been reported to activate their neighboring genes via a cis-mediated mechanism [28]. A few molecules can regulate gene expression at the epigenetic level via transcriptional interference and chromatin-mediated repression and histone modification, etc. [27,28]. Some lncRNAs are critical for genome stability via a topoisomerase complex [29]. Several lncRNAs act as “sponges” to compete with endogenous microRNAs(miRNAs), resulting the regulation on target genes of microRNAs (miRNAs) [27,30]. Further investigations on lncRNAs have supported their roles in a variety of key biological processes of plants similar to mammalian lncRNAs. In recent years, numerous lncRNAs responsive to biotic and abiotic stresses have been identified in many plant species [27,31,32,33,34]. For instance, a large number of lncRNAs responsive to nitrogen stresses, phosphate deficiency, osmotic and salt stress, cold stress, and drought stress etc., were mined in many plants via high-throughput sequencing technology [1,26,32,35,36], while many lncRNAs might be related to plant defense against fungi, bacteria, and virus pathogens [31,37,38,39,40,41]. In addition, the function of several plant lncRNAs involved in plant defense were experimentally verified. For example, lncRNA-ACOD1 was found to be essential for viral replication but not for viral entry [42]. The long intergenic noncoding RNA LINC-AP2 is negatively correlated with AP2 gene expression during Turnip crinkle virus infection in *Arabidopsis* [37]. LncRNA33732 in tomato was found to act as a positive regulator, enhance tomato resistance to *Phytophthora infestans* by induction of the expression of the respiratory burst oxidase gene, and increase the accumulation of H_2_O_2_ [43]. Tomato lncRNA23468 can compete with endogenous RNA to modulate *NBS-LRR* genes by decoying miR482b, thus regulating tomato resistance to *P. infestans* [39].

A large number of lncRNAs have been identified as being involved in drought, nitrogen stresses, and phosphate deficiency in maize [1,36,44]. As many as 18,165 high-confidence maize lncRNAs were defined from 749 RNA-Seq experiments across different tissues of inbred line B73 [45]. Although lncRNAs participate in the regulation of plant-microorganism interaction, there are no available reports on the characterization of lncRNA response to AM fungi as far as we know. In our previous study, several AM fungi-responsive miRNAs in maize roots were identified [46]. In this study, the same root samples were further analyzed by strand-specific RNA-Seq to identify AM fungi-responsive lncRNAs. The symbiosis related regulatory networks of differentially expressed lncRNAs-mRNAs-miRNAs were also constructed. The results can contribute to a better understanding of arbuscular mycorrhizal fungi symbiosis.

## 2. Results

### 2.1. Phenotypic Responses of Maize Seedlings to AM Fungus

**Figure 1:**
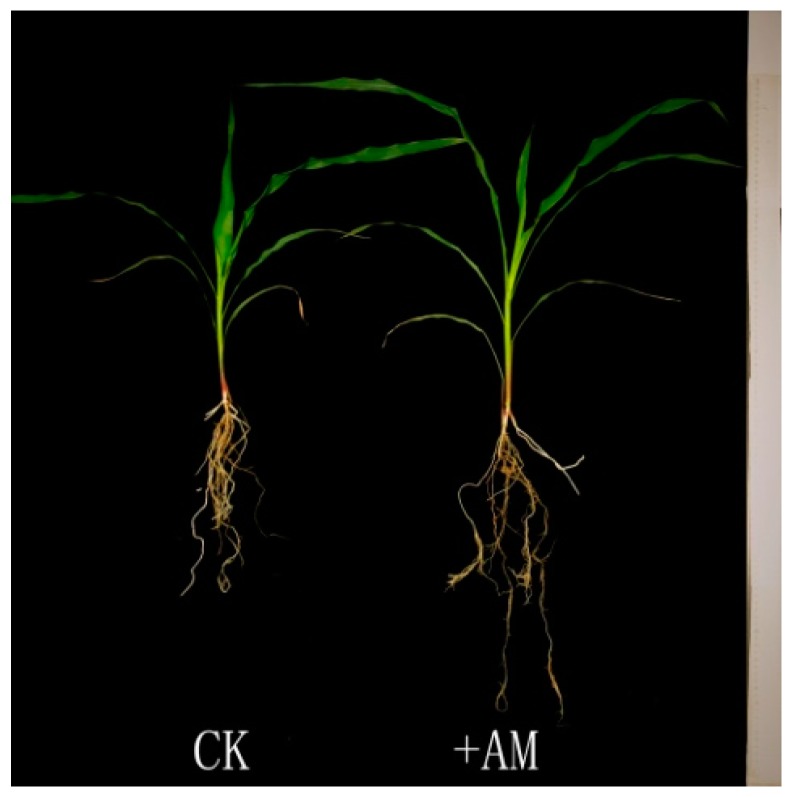
Influence of arbuscular mycorrhizal (AM) fungus on the growth of maize. CK, seedling without fungal inoculation; +AM, seedling inoculated with AM fungus.

The influence of AM fungus *Rhizophagus irregularis* (previously known as *Glomus intraradices*) on the growth of maize seedlings (inbred line B73) under phosphate deficiency was monitored. The maize seedlings inoculated with AM fungus were significantly taller than those in control (Figure 1, Table S1). The results suggested that AM fungus can significantly improve the growth of maize under phosphate-deficient conditions.

### 2.2. Ultra-Deep RNA Sequencing and Mapping onto Reference Genome

To obtain a comprehensive understanding of the maize transcriptome under fungal colonization, we isolated total RNAs from the maize roots with *R. irregularis* colonization. Roots without fungal inoculation were used as control. RNA-Seq libraries were constructed from the total RNA with ribosomal RNA (rRNA) depletion and sequenced by the paired-end method (150 bp × 2) with the Illumina HiSeq4000 platform. Sequencing of all samples yielded 315,494,666 raw paired-end 150-bp reads (SRA accession: PRJNA553580) (Table 1). After removal of low-quality bases and adaptors, ~86% of clean reads were mapped onto the *Z. mays* B73 genome using HISAT2 (Table 1). Table 1 shows that approximately 60% or more reads were uniquely mapped onto the reference genome, indicating that the result of alignment is reliable and can be used to identify differentially expressed protein coding genes (DEGs) and mine expressed lncRNAs.

### 2.3. Gene Ontology (GO) Term and Kyoto Encyclopedia of Genes and Genomes (KEGG) Pathway Enrichment Analyses of Identified DEGs

**Figure 2:**
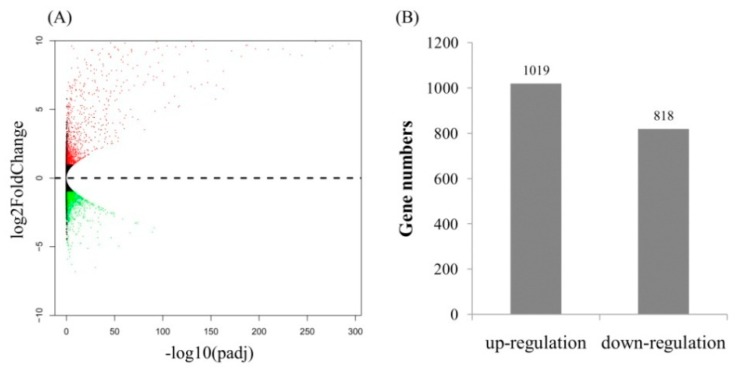
Differentially expressed genes (DEGs) between two samples. (**A**) Volcano plots; (**B**) Number of up-regulated and down-regulated genes.

**Figure 3:**
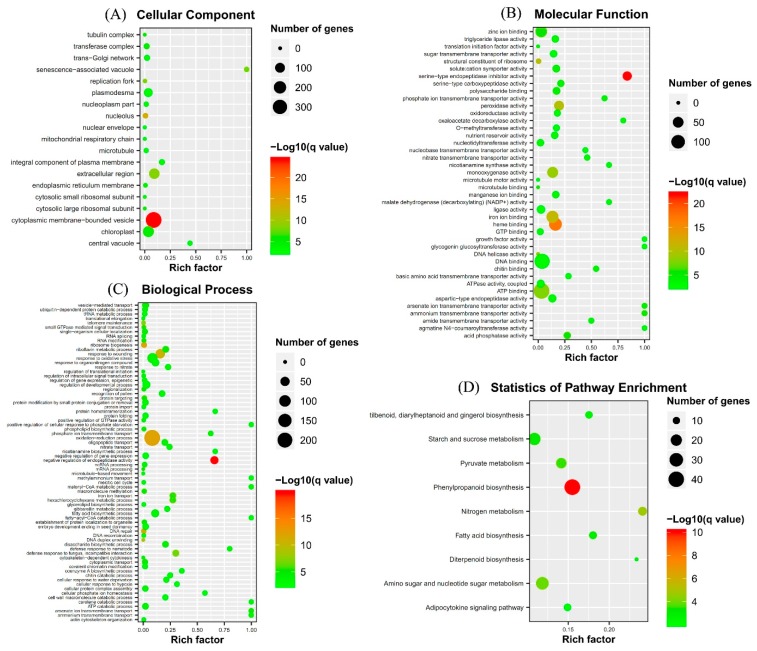
Gene Ontology (GO) term and Kyoto Encyclopedia of Genes and Genomes (KEGG) pathway enrichments of DEGs in response to AM fungus. (**A**) Cellular component, (**B**) Molecular function, (**C**) Biological process, and (**D**) KEGG pathway. Rich factor, percentage of genes in the background pathways.

The uniquely mapped reads were counted to identify DEGs. A total of 1837 protein coding genes were significantly differentially expressed between the fungal inoculated roots and control roots (*p* < 0.05) (Figure 2). Of those, 1019 genes were up-regulated, while 818 were down-regulated. Many AM fungi-responsive genes homologous to *MtPt4*, *STR*, *STR2*, and *MtFatM* were also included in the up-regulated genes, implying that the results of RNA-Seq is reliable. Enrichment analyses of DEGs showed that the GO terms were mainly enriched into the cytoplasmic membrane-bounded vesicle and senescence-associated vacuole in the cell component level (Figure 3). At the biological process level, the GO terms were mainly enriched into the oxidation−reduction process, the fatty-acyl-CoA catabolic process, the fatty acid biosynthetic process, the positive regulation of cellular response to phosphate starvation, and ammonium transmembrane transport (Figure 3). At the molecular function level, the GO terms were mainly enriched into ATP binding, DNA binding, and ammonium transmembrane transporter activity (Figure 3). KEGG pathway enrichment analyses showed that the DEGs were enriched into phenylpropanoid biosynthesis, nitrogen metabolism, fatty acid biosynthesis, and response to phosphate starvation, consistent with those in previous reports (Figure 3).

### 2.4. Identification and Characterization of Novel LncRNAs

**Figure 4:**
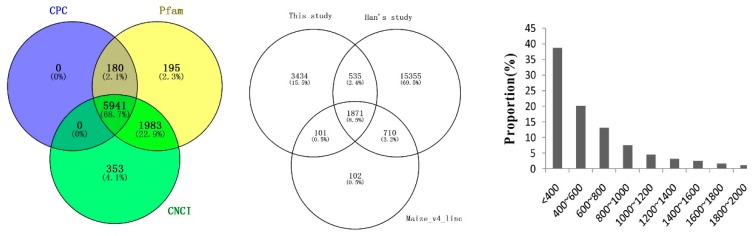
Venn diagram (left) of the number of noncoding transcripts, comparison of long noncoding RNAs (lncRNAs, middle) and distribution of lncRNAs (right).

A total of 198,964 transcripts were reconstructed from all of the six RNA-Seq datasets using StringTie. Tool gffcompare was used to obtain different and unknown transcripts by comparing known transcripts of the reference genome. The remaining 9070 unknown transcripts (>200 nt) were used to identify putative lncRNAs. The coding potential of the transcripts was predicted by CPC, CNCI and Pfam software. Of those, 5941 transcripts without coding potential by all three pipelines were considered as putative lncRNAs (Figure 4). After being compared with lncRNAs in the annotated maize genome (Version 4) and lncRNAs identified by Han et al. (2019), more than 3000 were newly discovered. The majority of the lncRNAs in this study were shorter than 1000 nt (Figure 4).

### 2.5. Putative Target Genes of Differentially Expressed lncRNAs (DELs)

**Figure 5:**
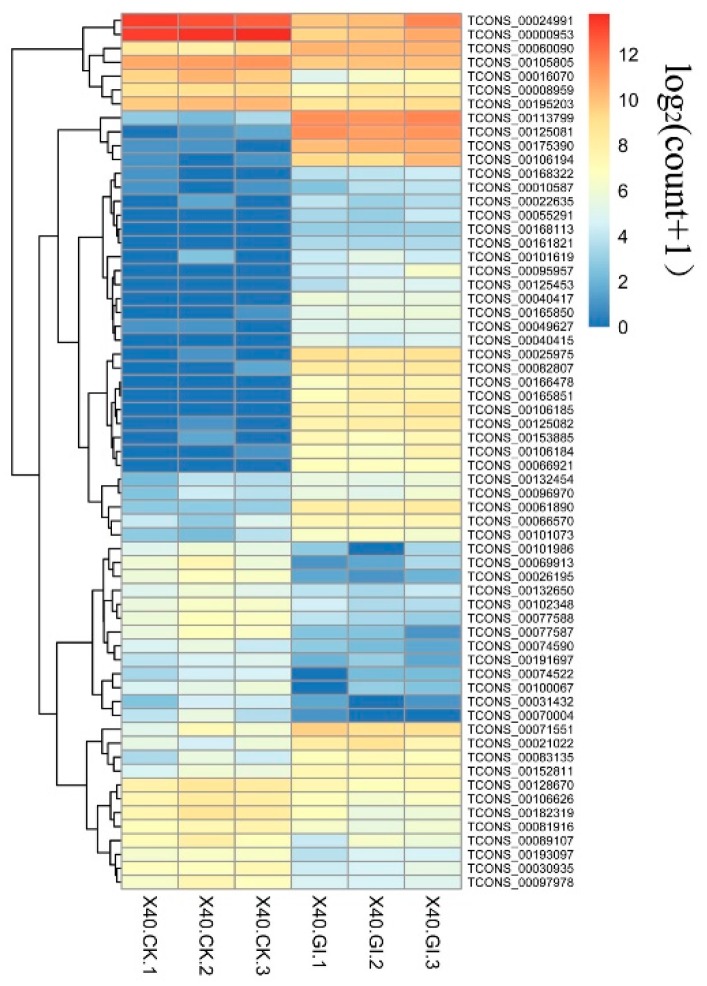
Heatmap of the differentially expressed lncRNAs. X40.CK, roots without fungal inoculation; X40.GI, roots inoculated *R. irregularis*. “1, 2, 3” represent biological replicates.

To identify differentially expressed maize lncRNAs between the AM fungal inoculation samples and control, lncRNAs with at least a 2.0-fold change in expression and *p* < 0.05 were considered to be differentially expressed. Among 5941 lncRNAs, a total of 63 lncRNAs were significantly differentially expressed (Figure 5). Of the DELs, 36 lncRNAs were up-regulated, while 27 were down-regulated.

A total of 266 protein-coding genes within the 100-kb region of DELs were predicted as cis-targeted genes (Table S2). Investigations have shown that lncRNAs can also regulate the target genes via interaction with miRNAs [44]. For the prediction of the target miRNAs, only the 16 differentially expressed miRNAs (DEMs) in the previous study [46] were used to predict the complementary miRNAs of lncRNAs. Of the DEMs, 15 miRNAs were predicted to be the complementary miRNAs of lncRNAs (Table S3). In total, 516 protein coding genes were predicted to be the targets of miRNAs (Table S4). A total of 778 protein-coding genes were predicted to be the target genes of DELs.

### 2.6. Regulatory Network of DELs

**Figure 6:**
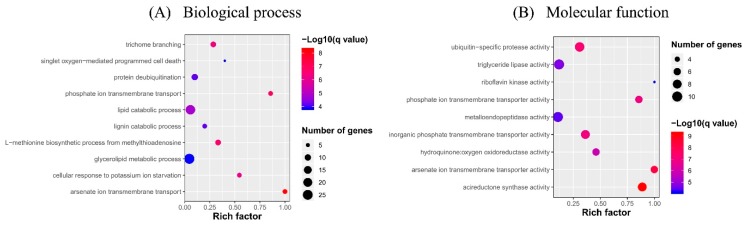
GO term enrichments of differentially expressed lncRNAs (DELs) in response to AM fungus. (**A**) Biological process, (**B**) Molecular function.

**Figure 7:**
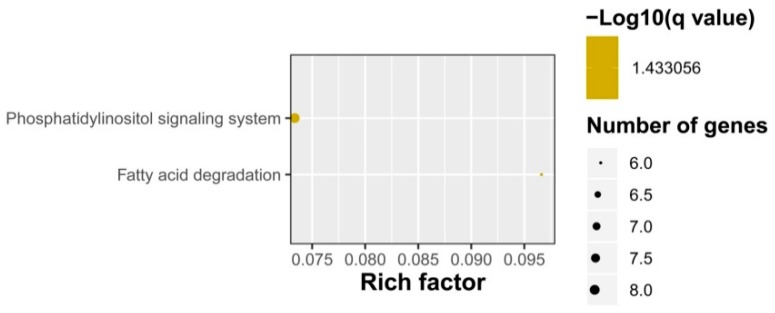
KEGG pathway enrichments of DELs in response to AM fungus.

Enrichment analysis of the putative targets of DELs showed that GO terms were enriched into phosphate ion transmembrane transport, cellular response to potassium ion starvation, lipid catabolic process, etc. in the biological process level, and triglyceride lipase activity, inorganic phosphate transmembrane transporter activity, phosphate ion transmembrane transporter activity, acireductone synthase activity, etc. in the molecular function level (Figure 6). No GO term was enriched in the cellular component category. KEGG pathway analysis showed that the target genes participated in fatty acid degradation and phosphatidylinositol signaling system (Figure 7). The phosphate ion transmembrane transport and fatty acid metabolic pathways play important roles in the interaction of plant and AM fungi, as reported in previous investigations [46,47,48].

**Figure 8:**
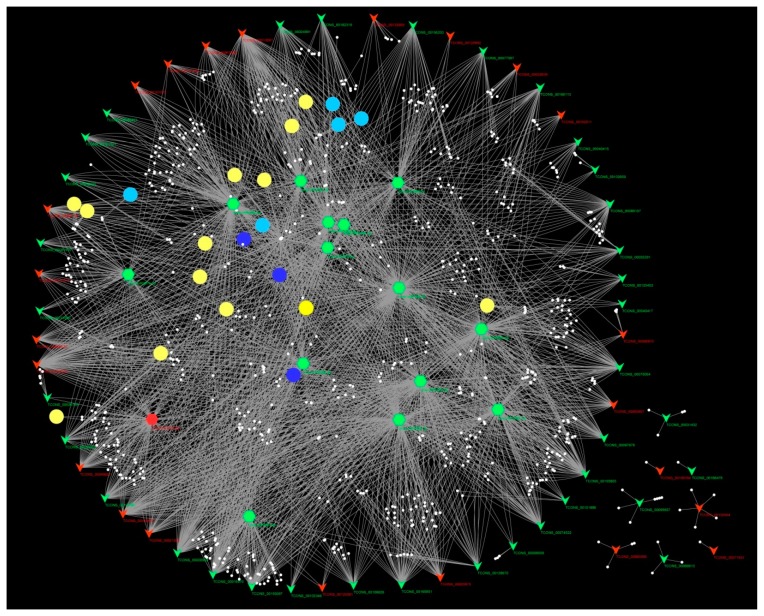
The potential regulating network of arbuscular mycorrhiza (AM)-responsive lncRNAs in maize root. Red inverted triangles represent up-regulated lncRNAs; green inverted triangles represent down-regulated lncRNAs; red hexagons represent up-regulated miRNAs; green hexagons represent down-regulated miRNAs; blue circles represent fatty acid metabolism-related pathways; cyan circles represent phosphate uptake-related pathways; yellow circles represent the rest of the enriched pathways; and white small circles represent the target genes.

The DELs, DEMs, putative target genes, and pathways were used to construct the regulatory network. It can be seen in the network that one DEL can interact with many DEMs, and one DEM can be regulated by many DELs (Figure 8). Majority of the DELs can regulate the target genes via miRNAs, indicating that the DELs might regulate genes involved in lipid metabolism and response to phosphate starvation via miRNAs (Figure 8).

### 2.7. Putative Vital DELs

Our previous investigation suggested that a few members of the miR399 family and the miR397 family (zma-miR395b-5p, zma-miR395h-5p, zma-miR397b-5p) should be involved in controlling the fatty acid metabolism and promoting lipid delivering from plants to AM fungi [46]. In the present study, several up-regulated lncRNAs e.g., TCONS_00083135, TCONS_00161821, TCONS_00153885, TCONS_00010587, TCONS_00165850, TCONS_00113799, and TCONS_00165851 were predicted to be the putative target mimics of zma-miR395b-5p, zma-miR395h-5p, and zma-miR397b-5p. In particular, TCONS_00113799, TCONS_00165850, and TCONS_00153885 were highly and almost specifically expressed in AM fungal colonized roots (Tables S4 and S5). Besides, a few up-regulated lncRNAs, i.e., TCONS_00113799, TCONS_00125081, TCONS_00060090, TCONS_00106194, and TCONS_00071551, were predicted to be the putative target mimics of miR399 family (Tables S4 and S5). These lncRNAs might be the key members involving in regulation of AM symbiosis.

### 2.8. Real-Time PCR Verification of Five DEGs and DELs

To validate the DEGs and DELs, we arbitrarily selected four up-regulated DELs and an AM fungi-responsive gene. Validation results show that the expression of fungal responsive gene *ZmPHT16–* was significantly up-regulated in AM fungal inoculation samples, in accordance with both RNA-Seq and real-time PCR (Figure S1), which indicated that the expression level of the coding genes was reliable. A similar expression pattern of the DELs was also observed between RNA-Seq and real-time PCR. All results indicated that the results of the RNA-Seq was reliable.

## 3. Discussion

It is estimated that 80–90% of land plants are colonized by AM fungi which can provide mineral nutrients to the plant and enhance plant resistance to adverse environments [10]. Although a large number of non-coding RNAs have been identified in animal and plant species [49,50,51,52], the profile of lncRNAs in plant root symbiosis with AM fungi has not been explored. In the present study, as many as 5941 lncRNAs were identified from the maize roots colonized with AM fungus. Han et al. (2019) identified 18,165 high-confidence lncRNAs from 749 RNA-Seq experiments across different tissues including roots of the maize inbred line B73 [45]. More than 2500 lncRNAs were annotated in the maize B73 genome (Version 4) [53]. Compared with the two lncRNAs libraries, more than 3000 lncRNAs were newly identified in this study, implying that many novel lncRNAs in roots might be transcribed by the stimulation of AM fungi. Some investigations concluded that over 100,000 lncRNAs have been defined in the human genome [54]. The expression of lncRNAs is more highly spatial and has more temporally restricted patterns than that of mRNAs [54], and some lncRNAs are only expressed in specific tissue, suggesting that lncRNAs should play different roles in regulating gene expression. In this study, 63 lncRNAs were differentially expressed in fungal colonized roots compared with the control. Further network investigation showed that the DELs can regulate the expression of several symbiosis pathways directly or indirectly. Some of the DELs might play important roles in the regulation or maintenance of symbiosis of plants with AM fungi.

The recognition and the maintenance of the symbiosis between plants and AM fungi is complex [13,55]. The signal molecules which mediate the recognition between plants and AM fungi have been identified [15,56]. Plant receptors that perceive the fungal signal molecules and many genes of the symbiosis signaling pathway have also been identified [15]. Once the plant and the fungi establish the symbiosis mycorrhizal relationship, frequent nutrient exchange takes place between the two species via the transporters, e.g., STR/STR2, PT4, AMT2;3, SUT2, HA1, etc., localized in the periarbuscular membrane of the symbiotic interface [13,15]. To maintain the symbionts, many genes are specifically expressed in higher levels during the interaction. Many plant transporters involving transport of different mineral and carbon nutrients, e.g., sugars, lipids, phosphorus, nitrogen, potassium, sulfate, and metal ions, etc., have been identified [13,47,48]. Several mycorrhiza-inducible genes, e. g., *Lotus japonicus* phosphate transporter 4 (*LjPT4*), *LjHA1* encoding H^+^-ATPase, STR involving in transport 2-monoacylglycerols, fatty acid biosynthesis genes (including *RAM2* encoding glycerol-3-phosphate acyltransferase), and *FatM* encoding an AMP-dependent synthetase and ligase are directly up-regulated by *AP2/ERF* family transcription factors CBX1 and/or WRI5a [10,24]. Chromatin Immunoprecipitation Sequencing (ChIP-seq) and experimental investigations showed that CBX1 or WRI5a can directly bind the conserved CTTC-containing promoter region of many mycorrhiza-inducible genes and activate the expression of the genes [23]. Although a few motifs (e.g., CTTC, TCTTGT) involving AM symbiosis have been identified, many genes without the motifs in their promoters were also significantly up-regulated in response to AM fungal colonization [24]. The facts suggest that the regulation mechanism of the expression of mycorrhiza-inducible genes is very complex. Non-coding RNAs or other regulation factors should participate the elaborate regulation of genes related to maintain mycorrhizal symbiont.

Bidirectional nutrient exchange must be regulated by both partners to maintain a stable symbiotic relationship, but the specific mechanism of regulating bidirectional nutrient exchange is still unclear [13]. Our previous investigation implied that a few differentially expressed miRNAs might coordinately regulate the exchange of lipids and phosphate between plant and AM fungi [46] The further regulatory network in this study indicated that lncRNAs might also participate the dynamic regulation exchange of nutrients and fatty acids directly or indirectly during the mycorrhizal symbiosis. LncRNAs regulate their targets in cis or trans manner at multiple levels through diverse mechanisms [32,39,44,57]. They may regulate mRNA processing and transcription mechanisms, e.g., splicing, editing, etc., and activate their neighboring genes using a cis-mediated mechanism [27]. Some lncRNAs act as miRNA target mimics, while other lncRNAs are precursors of miRNAs and small interfering RNA (siRNAs). LncRNAs, as part of plant defense mechanisms, are crucial for responses to phytopathogens revealed by recent investigations. For example, expression of the At2g30770 gene and its lncNAT were coordinately regulated in response to *Fusarium oxysporum* infection [58]. Another investigation on TYLCV-resistant tomato line CLN2777A observed several differentially regulated lncRNAs in response to tomato yellow leaf curl virus (TYLCV), and lncRNAs positively regulated the expression of miRNA targeting protein-coding genes through miRNA target mimicry [43]. In the present study, the majority of the DELs might regulate the expression of the target genes via miRNA target mimicry, implying that the DELs and DEMs should coordinately regulate the nutrient exchange between plant and AM fungi. Specifically, up-regulated lncRNAs TCONS_00113799, TCONS_00165850, and TCONS_00153885 were predicted to be the putative target mimics of zma-miR395b-5p, zma-miR395h-5p, and zma-miR397b-5p. The three lncRNAs might mediate the expression of genes involving fatty acid metabolism via competition with putative target miRNAs [46]. In addition, TCONS_00113799, TCONS_00125081, TCONS_00060090, TCONS_00106194, and TCONS_00071551 can competitively bind to miRNAs in miR399 family which are involved in phosphate starvation response [59,60], suggesting that these non-coding RNAs might participate in the regulation phosphate starvation response via the miR399 family. The rice cis-natural antisense RNA (cis-NAT*_PHO1;2_*) is a translational enhancer which promotes *PHO1;2* translation and contributes to phosphate homeostasis and plant fitness [61]. Sequence similarity analysis showed no similar sequence to the three lncRNAs in NCBI database. The results of no similarity between cis-NAT*_PHO1;2_* and the up-regulated lncRNAs as putative targets of miR399 family in this study implied that many different lncRNAs are involved in regulation of the uptake and/or transfer of phosphate. Several lncRNAs are shared in the regulatory network of fatty acid metabolism and phosphate starvation response, suggesting that the regulation mechanism is complex, and many lncRNAs might be involved in the regulation of bidirectional nutrient exchange between plant and their fungal partners.

## 4. Materials and Methods

### 4.1. Plant Symbiosis with AM Fungus

*Z. mays* (Inbred line B73) seeds were surface sterilized, germinated, inoculated with AM fungus *R. irregularis* DAOM-197198 [62], and watered as previously described [46]. The fungal strain was provided by Bank of Glomales in China. The roots of maize seedlings at 40 days after inoculation were used for strand-specific RNA-Seq. Three repeats were performed.

### 4.2. RNA Extraction and Total RNA-Seq Sequencing

Total RNA was isolated from maize roots using RNiso Plus reagent (Takara, Dalian, China), and then was treated with RNAase-free DNAase I according to the manufacturer’s protocols. The quality and quantity of total RNAs were analyzed via an Agilent 2100 and a Nano-Drop 2000c instrument (Thermo Scientific, waltham, MA, USA). Each total RNA was treated with the Ribo-Zero™ rRNA Removal Kit (Illumina, Redwood City, CA, USA) to remove rRNAs. The TruSeq RNA Sample Prep Kit v2 (Illumina, Redwood City, CA, USA) was used to construct strand-specific RNA-sequencing libraries according to the manufacturer’s instructions. The fragments in an expected size range were sequenced using an Illumina Hiseq 4000 platform by Lc-bio, Hangzhou, China.

### 4.3. Pipelines for Differentially Expressed Genes (DEGs) and Novel LncRNAS Identification

FastQC was first applied to evaluate the quality of the raw Illumina reads. Trimmomatic (v0.33) was used to trim the paired-end raw reads and remove low-quality base-calls and adaptor sequences with parameters 2:30:10 LEADING:3 TRAILING:3 SLIDINGWINDOW:4:15 HEADCROP:12 MINLEN:36. Cleaned reads were mapped to the genome of *Z. mays* B73 (Version 4; http://plants.ensembl.org) with HISAT2 (v2.1.0) [63]. Uniquely mapped reads were used to quantify the raw counts of known genes using HTSeq (v0.9.1) [64]. DEGs were calculated by DESeq (v1) in R using the parameters: *p* < 0.05 and fold change >2 [65].

After the alignment, StringTie was carried out to assemble reads into transcripts. Transcripts from all assemblies were merged to generate a unique transcript assembly by Stringtie (v1.3.3) [63]. The new assembled transcripts were compared with known protein encoding transcripts to obtain different and unknown transcripts by using the gffcompare (v0.9.9c) [63]. The remaining unknown transcripts (>200 nt) were used to identify putative lncRNAs. The coding potential of the transcripts was predicted by CPC, CNCI and Pfam software. A transcript was considered as candidate lncRNAs if the coding potentials are scored to be less than −1 by all the three software. The differentially expressed lncRNAs (DELs) were also calculated by DESeq using the parameters: *p* < 0.05 and fold change >2.

### 4.4. Prediction of Putative Cis- and Trans-targets of DELs

Many investigations have showed that lncRNAs can regulate the expression of neighboring protein-coding genes via epigenetic modification and/or transcriptional co-activation/repression. The protein-coding genes franking 100-kb regions of the lncRNAs were selected as the cis-target genes. The complementary miRNAs of lncRNAs were predicted using RIsearch (v2.0) [64] with minimum free energy of –20 kcal/mol. The target genes of miRNAs were predicted by the psRNATarget algorithm (accessed online: http://plantgrn.noble.org/psRNATarget).

### 4.5. GO and KEGG Pathway Enrichment Analyses

The genes of *Z. mays* (Version 4) were annotated against GO and KEGG database, respectively. Fisher’s exact test was used to obtain enriched functional terms of DEGs. The putative target genes of DELs were also used to enrich the GO terms and KEGG pathways.

### 4.6. Construction of the Regulatory Network

The regulatory network was constructed as previously described [46]. Briefly, the relationship pairs between lncRNA and miRNA, between miRNA and the target gene, between the target gene and GO terms, and between the target gene and the KEGG pathway were used as the edges during network construction. The data of the relationship pairs between lncRNA and miRNA, between miRNA and the target gene, between the target gene and GO terms, and between the target gene and the KEGG pathway were imported into Cytoscape (version 3.7.1) [66]. The definition data of lncRNA, miRNA, the target gene, GO terms, and the KEGG pathway were also imported into Cytoscape as nodes. A network was visualized and adjusted manually with Cytoscape.

### 4.7. Validation of DEGs and DELs by Quantitative RT-PCR

Five arbitrarily up-regulated DELs and AM fungi-responsive genes were selected for quantitative RT-PCR, and the β-actin gene was used as the endogenous control. Total RNA was reverse transcribed into cDNA using a Reverse Transcription kit (Takara, Dalian, China) according to the manufacturer’s protocol. The 20 µL reaction system consisted of 120–150 ng cDNA, gene-specific primers (0.5 µL, 10 µmol L^−1^) (Table S6), and 5 × SYBR Green Master Mix (10 µL). The real-time quantitative PCR program was set to the following procedures: 95 °C for 10 min, followed by 40 cycles of 95 °C for 15 s and 60 °C for 1 min. Relative expression levels were calculated using the 2^−ΔΔ*C*t^ method. Three biological replicates for each sample were used for quantitative RT-PCR.

## 5. Conclusions

A total of 5941 lncRNAs were identified, and more than 3000 were newly discovered. Of those, 63 lncRNAs were differentially expressed. Thirty-six lncRNAs were up-regulated, while 27 were down-regulated in maize root in response to fungus *R. irregularis* inoculation. The differentially expressed lncRNAs might participate in the regulation of bidirectional nutrient exchange between plant and AM fungi via miRNA target mimicry. The function of DELs needs to be experimentally verified via over-expression or knock-out methods in the future. The results of this study could contribute to a better understanding of the regulatory network between plants and AM fungi.

## Figures and Tables

**Table 1:** Statistics of RNA-Seq read mapping results.

Sample.	Raw Reads	Number of Clean Reads	Uniquely Mapped Reads Number	Uniquely Mapped Reads (%)	Reads Mapped to Multiple Loci (%)	Reads Unmapped: Too Short (%)
40-CK-1	43,293,767	37,470,252	23,577,438	62.92	5.18	31.90
40-CK-2	53,402,529	46,776,062	32,169,682	68.77	4.52	26.71
40-CK-3	55,730,952	49,755,232	32,920,777	66.17	4.89	28.95
40-GI-1	51,811,515	44,618,959	26,549,282	59.50	4.52	35.98
40-GI-2	57,804,926	49,070,512	31,196,619	63.58	4.06	32.36
40-GI-3	53,450,977	46,286,135	30,037,248	64.89	4.10	31.01

Reads were counted as pairs. 40-CK, roots without fungal inoculation; 40-GI, roots inoculated *R. irregularis*. “-1, -2, -3” represent biological replicates.

## Supplementary Materials

Supplementary Materials can be found at https://www.mdpi.com/1422-0067/20/18/4491/s1.
