# Supplementary material for: Identification of Long Non-Coding RNAs and the Regulatory Network Responsive to Arbuscular Mycorrhizal Fungi Colonization in Maize Roots
Source: Int J Mol Sci. 2019 Sep 11;20(18):4491. doi: 10.3390/ijms20184491 (PMC6769569; doi:10.3390/ijms20184491)
Supplement: Supplementary file 1 [file ijms-20-04491-s001.zip › ijms-568110-SI/Supplementary File(s) new/Supplementary Table 1 taller.docx]

**Supplementary Table 1.** The taller of seedling.

| Control | Treatment |
| --- | --- |
| 53.67±5.13 | 76.83±7.97* |

Note: Treatments, roots inoculated with AM fungus; Control, roots without AM fungus inoculation. **p*<0.05.
