# Supplementary material for: Identification of Long Non-Coding RNAs and the Regulatory Network Responsive to Arbuscular Mycorrhizal Fungi Colonization in Maize Roots
Source: Int J Mol Sci. 2019 Sep 11;20(18):4491. doi: 10.3390/ijms20184491 (PMC6769569; doi:10.3390/ijms20184491)
Supplement: Supplementary file 1 [file ijms-20-04491-s001.zip › ijms-568110-SI/Supplementary File(s) new/Supplementary Table 6. primers.docx]

**Supplementary Table 6.** Primers used in this study.

| Coding sequence ID | Primer sequence |
| --- | --- |
| *TCONS_00025975* | TCAGGAGCAGCATCACATCT |
|  | CGCTAGAAGAACGCACGAAC |
| *TCONS_00125081* | AAGAGGATGGGATCGACGAC |
|  | CCCCTGCCTAGATACATGCA |
| *TCONS_00165851* | TAAAACGTTCCAGCAGCCAC |
|  | GATGCAACCACCGACTCATC |
| *TCONS_00175390* | GGGGTCTCGTTCAAATCCAC |
|  | TATGCCGGACAAGACCAGTT |
| *ZmPHT1-6* | ACGTGGTTCCTTCTCGACAT |
|  | CCTTGAGGGCGTTTATGTCG |
