# Supplementary figures and images for: Identification of Long Non-Coding RNAs and the Regulatory Network Responsive to Arbuscular Mycorrhizal Fungi Colonization in Maize Roots
Source: Int J Mol Sci. 2019 Sep 11;20(18):4491. doi: 10.3390/ijms20184491 (PMC6769569; doi:10.3390/ijms20184491)

**
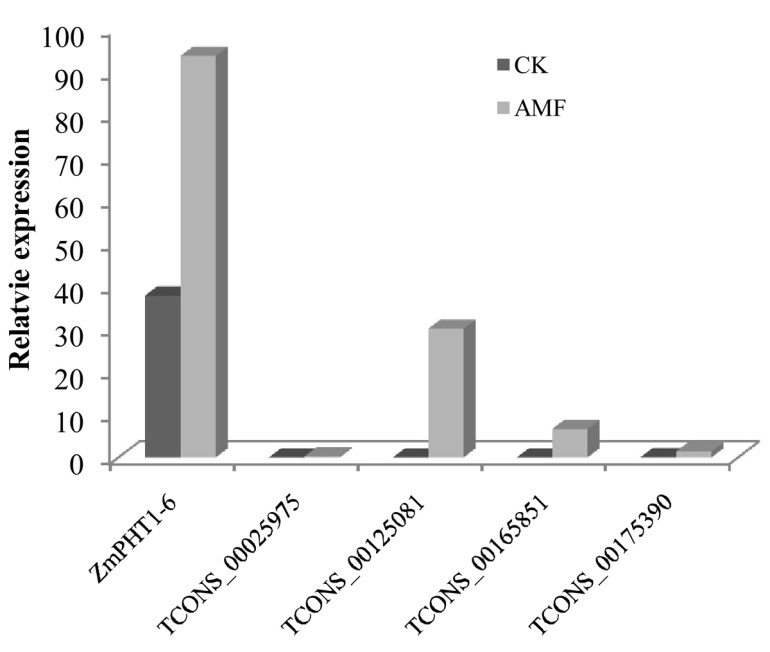
**

**Supplementary Figure 1.** Real-time PCR verification of five up-regulated DEGs and DELs.

Supplement: Supplementary file 1 [file ijms-20-04491-s001.zip › ijms-568110-SI/Supplementary File(s) new/Supplementary Figure 1.docx]
